# The Efficacy and Safety of Apremilast in the Management of Psoriatic Arthritis: A Systematic Review and Meta-Analysis

**DOI:** 10.7759/cureus.55773

**Published:** 2024-03-08

**Authors:** Renad F Alharthy, Joud M Alharthy, Razan O Bawazir, Renad I Katib, Fayez S Alharthy

**Affiliations:** 1 College of Medicine, King Saud Bin Abdulaziz University for Health Sciences, Jeddah, SAU; 2 College of Medicine, King Abdullah International Medical Research Center, Jeddah, SAU; 3 College of Medicine, King Abdullah International Medical Research Center, Jeddah, Saudi Arabia, Jeddah, SAU; 4 Internal Medicine/Rheumatology, King Abdulaziz Medical City, Jeddah, SAU; 5 Internal Medicine/Rheumatology, College of Medicine, King Saud Bin Abdulaziz University for Health Sciences, Jeddah, SAU

**Keywords:** medicine, meta-analysis, pde-4 inhibitors, psoriatic arthritis, apremilast

## Abstract

Psoriasis is a chronic autoimmune inflammatory skin disease that is associated with other conditions, one of them being psoriatic arthritis (PsA). Apremilast, a phosphodiesterase-4 inhibitor, displayed promising results in multiple trials for patients with PsA. This systematic review and meta-analysis aims to showcase its efficacy and safety when compared to placebo. Preferred Reporting Items for Systematic Reviews and Meta-Analysis (PRISMA) was adopted after registration on the International Prospective Register of Systematic Reviews (PROSPERO: CRD42023476245). Four databases were systematically searched from their inception until October 25, 2023. As a result, five randomized controlled trials were included with 1,849 participants, after thorough screening. The primary efficacy endpoint evaluated in this meta-analysis was the American College of Rheumatology Response Criteria 20 (ACR20). The results significantly favored apremilast (risk ratio [RR] = 1.92, 95% confidence interval [CI] 1.66-2.21; *P* < 0.00001; *I*^2^= 0%) as opposed to placebo. Similarly, secondary efficacy endpoints, ACR50 (RR = 2.34, 95% CI 1.79-3.06; *P* < 0.00001; *I*^2^ = 0%), ACR70 (RR = 2.89, 95% CI 1.62-5.18; *P* = 0.0003; *I*^2^ = 0%), and the Health Assessment Questionnaire and Disability Index (HAQ-DI; standardized mean difference [SMD] = -0.26, 95% CI -0.34 to -0.17; *P* < 0.00001; *I*^2^ = 0%) were also in significant favor of apremilast. However, apremilast had a higher occurrence of gastrointestinal adverse events than placebo (RR = 1.21, 95% CI 1.12-1.30; *P* < 0.00001; *I*^2^ = 19%). To conclude, apremilast shows promising efficaciousness with some nonserious side effects when compared to placebo, but further trials are needed for comparison with other management lines.

## Introduction and background

Psoriatic arthritis (PsA), which manifests in approximately 20% of patients with psoriasis [1], is a chronic, autoimmune inflammatory disease resulting in stiffness and swelling in one, two to three, or more than three joints [2]. Traditionally, treatment for PsA has included conventional disease-modifying antirheumatic drugs (DMARDs) and non-steroidal anti-inflammatory drugs (NSAIDs). DMARDs are immunosuppressive and immunomodulatory agents, and despite there being scanty evidence to support their efficacy, especially as a monotherapy, methotrexate is generally recommended as a first-line therapy [3]. Recently, several management guidelines have been updated to include clinical phenotypic differences, including axial involvement, enthesitis, and dactylitis [4].

One class of drugs included in the latest Group for Research and Assessment of Psoriasis and Psoriatic Arthritis (GRAPPA) guidelines [5] is phosphodiesterase-4 inhibitors, with apremilast being particularly notable. Apremilast, approved by the U.S. Food and Drug Administration in March 2014 for the treatment of PsA [6], is recommended for concurrent use with csDMARDs. Apremilast works by downregulating the inflammatory pathway [7]. It does this by increasing cyclic adenosine 3',5'-monophosphate levels, thus reducing pro-inflammatory agents' expression and increasing the production of anti-inflammatory agents [8]. Several randomized controlled trials (RCTs) have been published that compare the safety and efficacy of apremilast in patients previously exposed to DMARDs, as opposed to those taking a placebo [9-13]. In this study, we aimed to compile the results of those trials in a systematic review. We used a Population, Intervention, Control, Outcome (PICO) model as our principal inclusion criterion. Therein, we screened RCTs whose population was adults who had been diagnosed with PsA based on any known criteria. The intervention was apremilast; the control was a placebo; and the primary outcome being measured was the American College of Rheumatology score, or ACR20 [14]. We also measured several secondary outcomes: the Health Assessment Questionnaire (HAQ) and Disability Index (DI) [15], ACR50 [16], and ACR70 [17] and reported serious adverse events, all adverse events, and mortality for safety indexing. To our knowledge, there have been no previous systematic reviews of this kind.

The ultimate goal of this study is to create a reference that compiles all existing literature on the safety and efficacy of apremilast and assess the literature for validity, bias, and other confounding variables. This systematic review and meta-analysis is part of a larger effort to expand treatment for PsA and tailor it to each patient’s needs.

## Review

Methodology

We registered our title and idea on the International Prospective Register of Systematic Reviews (PROSPERO: CRD42023476245) before applying for the initial search. The Preferred Reporting Reporting Items for Systematic Reviews and Meta-Analysis (PRISMA) checklist was strictly used [18]. Furthermore, no ethical approval or consent was needed since all data are available online. Patients' consent was collected before the initiation of the trials in all included RCTs.

Eligibility measures

This systematic review and meta-analysis only encompasses RCTs that compared apremilast and placebo. The inclusion criteria included adult patients (aged 18+ years) diagnosed with PsA or who met the Classification Criteria for Psoriatic Arthritis (CASPAR) criteria, patients on a controlled dose of concurrent drugs (prednisone ≤10 mg/day, methotrexate ≤25 mg/week, and NSAIDs before baseline stratification), and patients who had never been exposed to apremilast. In addition, both patients who were tumor necrosis factor (TNF) inhibitors naive or intolerant were included. On the other hand, RCTs that exclusively included DMARD-naive patients were excluded to not skew the results. Finally, any RCTs that did not align with our primary outcome, ACR20, were excluded.

Search strategy

The following databases were systematically searched: Medline, Web of Science, ClinicalTrials.gov, and CENTRAL (Cochrane Central Register of Controlled Trials). The search was conducted from the inception of each database until October 25, 2023, with no filter on language or otherwise. Moreover, references to the included RCTs were reviewed for other studies. The search strategy is provided in Table 1.

**Table 1:** Search strategy. PDE4 inhibitor, phosphodiesterase 4 inhibitor; CC-10004, apremilast; TS, topic

ClininicalTrials. gov
1) Condition or Disease: Psoriatic Arthritis OR Arthritic Psoriasis
2) Other terms: Psoriasis Arthropathy
3) Intervention/Treatment: Apremilast OR Phosphodiesterase 4 inhibitor OR PDE4 inhibitor OR CC-10004
PubMed
(((((apremilast) OR (phosphodiesterase 4 inhibitor)) OR (PDE4 inhibitor)) OR (PDE-4 inhibitor)) OR (CC-10004)) AND (((((((((arthritis, psoriatic[MeSH Terms]) OR (arthritic psoriasis[MeSH Terms])) OR (psoriatic arthritis[MeSH Terms])) OR (psoriasis, arthritis[MeSH Terms])) OR (psoriasis arthropathica[MeSH Terms])) OR (psoriasis arthropathy[MeSH Terms])) OR (arthropathies, psoriatic[MeSH Terms])) OR (arthropathy, psoriatic[MeSH Terms])) OR (psoriatic arthropathies[MeSH Terms])) Filter: Randomized Control Trials
Web of Science
1) (((TS=(arthritic psoriasis)) OR TS=(psoriatic arthritis)) OR TS=(psoriasis arthropathy)) OR TS=(psoriasis arthritis)
2) ((((TS=(apremilast)) OR TS=(phosphodiesterase 4 inhibitor)) OR TS=(PDE4 inhibitor)) OR TS=(CC-10004)) OR TS(otezla)
3) 1 AND 2 AND Clinical trial
CENTRAL
1) exp Arthritis, Psoriatic/
2) Arthritis, Psoriatic$.mp.
3) Arthritic psoriasis.mp.
4) 1 or 2 or 3
5) Apremilast.mp.
6) exp Phosphodiesterase 4 inhibitor/
7) CC-10004.mp.
8) Otezla.mp.
9) Phosphodiesterase 4 inhibitor$.mp.
10) 5 or 6 or 7 or 8 or 9
11) 4 and 10
Filter: Clinical Trial

Study selection and data extraction

Studies were independently selected on three rounds of title, abstract, and full-text screening based on the inclusion criteria by four reviewers. Disagreements were discussed and resolved with the senior professor (FA). Data extraction was executed independently by two separate reviewers on an Excel sheet, which included study design, duration of exposure, study arms, sample size, demographics, study population, follow-up, and concurrent treatment.

Endpoints

The outcomes of this study were selected based on changes in rheumatoid arthritis symptoms and functionality. The primary efficacy endpoint is ACR20. ACR50, ACR70, and mean change from baseline in the HAQ-DI are the secondary measured efficacy outcomes. Dichotomous outcomes in ACR20/50/70 were provided in most RCTs. However, one RCT provided the percentage of patients. As a result, the percentages were reformed into dichotomous outcomes by multiplying them by the referred sample size and dividing by 100, while considering the associated *P*-value. All efficacy outcomes were assessed at either week 16 or 12. Finally, the safety index was identified using the occurrence of any adverse events (AEs), serious AEs (SAEs), and mortality as dichotomous outcomes.

Meta-analysis

Data analysis was conducted using Review Manager Web (RevMan Web). The data source was entered manually, and the analysis model utilized the random effects criteria, with *P* < 0.05. For dichotomous outcomes (ACR20, ACR50, ACR70, AEs, SAEs, and mortality), the inverse variance statistical method and the risk ratio (RR) effect measure were applied with a confidence interval (CI) of 95%. For continuous outcomes (HAQ-DI), the inverse variance statistical method and standard deviation (SD) mean difference effect measure were applied with a 95% CI. Studies that used the continuous outcomes of HAQ-DI aside with a standard error (SE) were converted to SD by multiplying it by the square root of its corresponding sample size. Any statistical heterogeneity was evaluated by chi^2^ or *I*^2^. Data were subgrouped into three categories based on the dose of apremilast and treatment endpoint in comparison to placebo. The first subgroup included apremilast 30 mg BID (twice a day) evaluated at week 16. The second subgroup encompasses apremilast 20 mg BID assessed at week 16. The third subgroup contained apremilast 20 mg BID appraised at week 12.

Results

**Figure 1:**
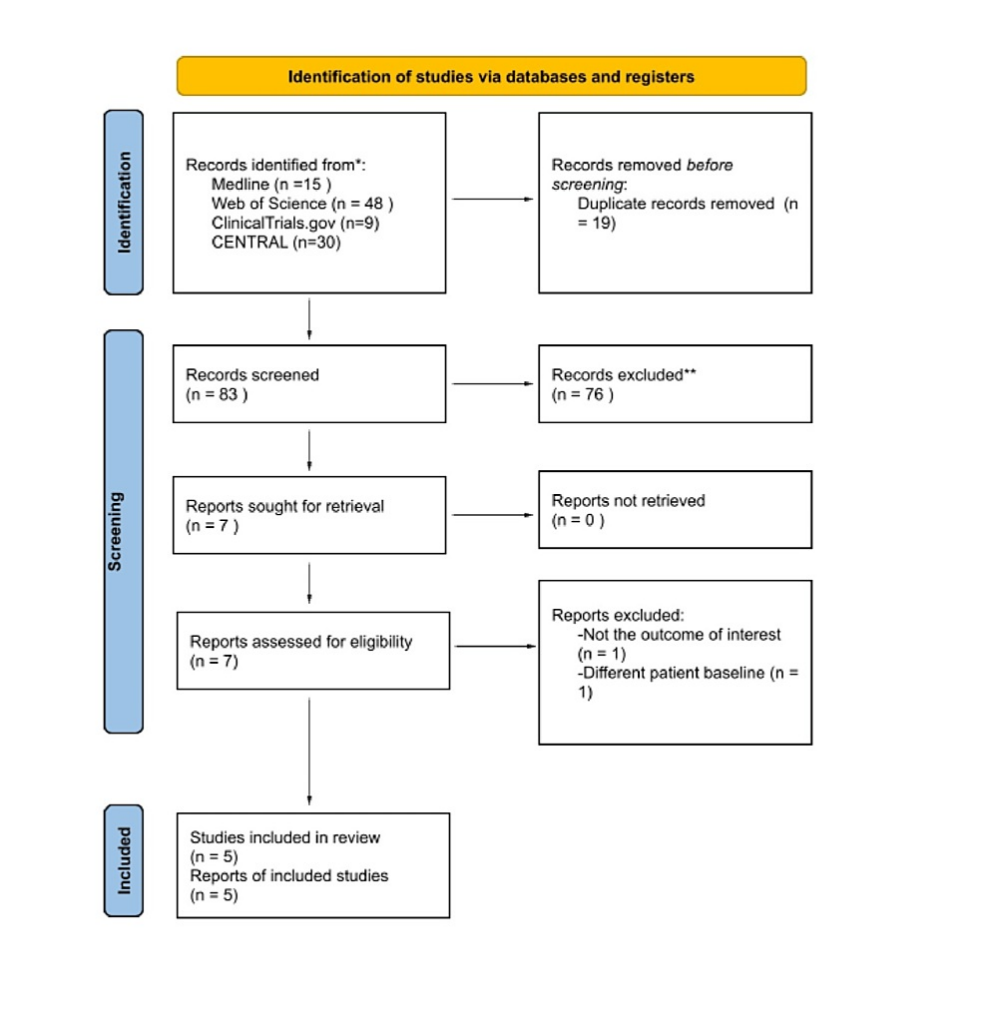
Study flowchart as per the Preferred Reporting Items for Systematic Reviews and Meta-Analysis (PRISMA) criteria. CENTRAL, Cochrane Central Register of Controlled Trials; RCT, randomized controlled trial

Following the preliminary search, 102 studies were downloaded. After 19 duplicates were excluded, 83 records were screened by the title and abstract, and 76 articles were excluded. Only seven RCTs were included in the full-text review. Two RCTs were excluded for not matching our inclusion criteria; as a result, five RCTs were included in our systematic review and meta-analysis (Figure 1).

Trial baseline characteristics

The included trials cumulatively assessed 1,849 patients (Table 2). The sample size of patients in apremilast 30 mg, apremilast 20 mg, and placebo subgroups consisted of 608, 596, and 676, respectively. Regarding gender, females overall comprised the majority (984, 53.2%) of patients. Age baseline was provided as mean and SD in all trials. Moreover, age was similar in most trials. 

**Table 2:** Baseline characteristics of included trials. CT.gov, ClinicalTrials.gov registry; BID, twice a day; SD, standard deviation

CT.gov identifier	Study arms	Number of participants (started)	Number of participants (completed)	Age group, mean (SD)	Females, *n* (%)
NCT01925768	30 mg apremilast BID	110	91	50.7 (12.22)	58 (52.7%)
	Placebo	109	101	48 (13.75)	65 (59.6%)
NCT01172938	30 mg apremilast BID	168	154	51.4 (11.72)	92 (54.8%)
	20 mg apremilast BID	168	158	48.7 (10.99)	83 (49.4%)
	Placebo	168	158	51.1 (12.13)	80 (47.6%)
NCT01212757	30 mg apremilast BID	163	149	50.5 (11.2)	95 (58.6%)
	20 mg apremilast BID	163	151	50.9 (11.82)	95 (58.3%)
	Placebo	162	148	51.2 (10.97)	85 (53.5%)
NCT01212770	30 mg apremilast BID	167	156	49.9 (11.38)	88 (52.7%)
	20 mg apremilast BId	169	157	49.6 (12.10)	90 (53.3%)
	Placebo	169	156	49.5 (11.64)	91 (53.8%)
NCT00456092	20 mg apremilast BID	69	55	50.9 (12.58)	26 (37.7%)
	Placebo	68	50	51.1 (10.80)	36 (52.9%)

Risk of bias

**Figure 2:**
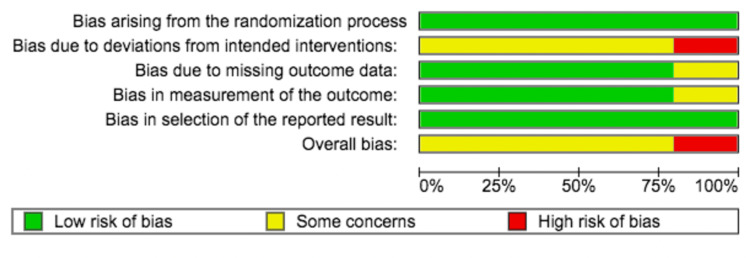
Risk-of-bias graph.

The revised Cochrane risk-of-bias tool for RCTs (Rob 2) was utilized in the included articles [19]. Two reviewers independently and together rated each domain as low risk, some concerns, or high risk (Figure 2). Disagreements were resolved through discussion. Potential publication bias was not achievable as the studies included were less than 10.

Efficacy outcomes

ACR20

**Figure 3:**
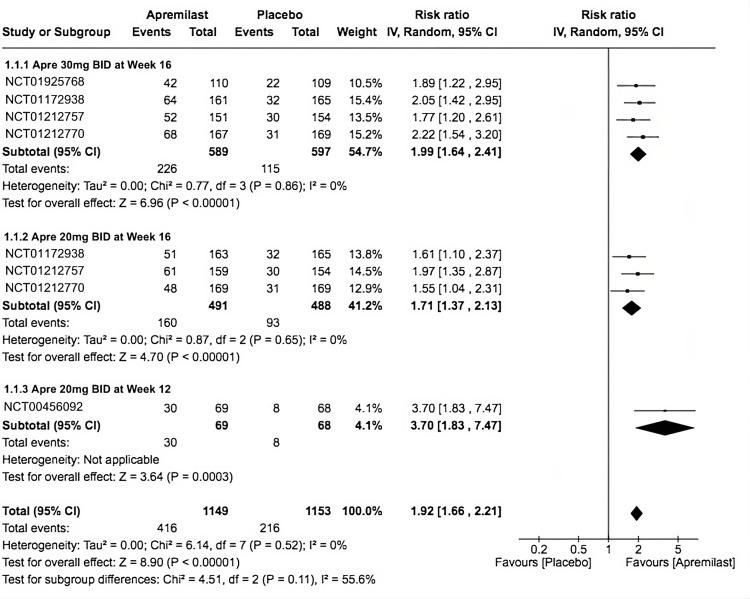
Forest plot of ACR20. Sources: [9-13]. CI, confidence interval; IV, inverse variance; RR, risk ratio; BID, twice a day; ACR20, American College of Rheumatology Response Criteria 20

Our primary outcome measures the improvement in the tender joint count, swollen joint count, and additional measures of 20%. The overall RR = 1.92 (95% CI 1.66-2.21) significantly favored apremilast with heterogeneity of *I*^2 ^= 0 and *P* < 0.00001. The third subgroup had a higher occurrence when compared to placebo (RR = 3.7, 95% CI 1.83-7.47; *P* = 0.0003, *I*^2 ^= not applicable), followed by the first subgroup (RR = 1.99, 95% CI 1.64-2.41; *P* < 0.00001; *I*^2^ = 0%), and finally, the second subgroup (RR = 1.71, 95% CI 1.37-2.13; *P* < 0.00001; *I*^2^ = 0%; Figure 3).

ACR50

**Figure 4:**
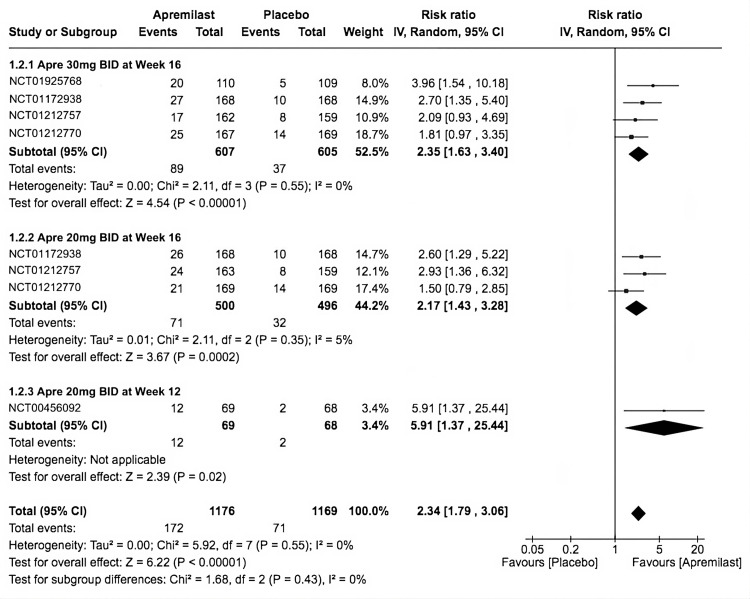
Forest plot of ACR50. Sources: [9-13]. CI, confidence interval; IV, inverse variance; RR, risk ratio; BID, twice a day; ACR50, American College of Rheumatology Response Criteria 50

The included trials showed more occurrences of ACR50 at the provided time frames in significant favor of apremilast (RR = 2.34, 95% CI 1.79-3.06; *P* < 0.00001; *I*^2^ = 0%). Similarly, the third subgroup had a higher occurrence when compared to placebo (RR = 5.91, 95% CI 1.37-25.44; *P* = 0.02; *I*^2^= not applicable), followed by the first subgroup (RR = 2.35, 95% CI 1.63-3.40; *P* < 0.00001; *I*^2^ = 0%). Finally, the second subgroup significantly scored (RR = 2.17, 95% CI 1.43-3.28; *P* = 0.0002; *I*^2^ = 5%; Figure 4).

ACR70

**Figure 5:**
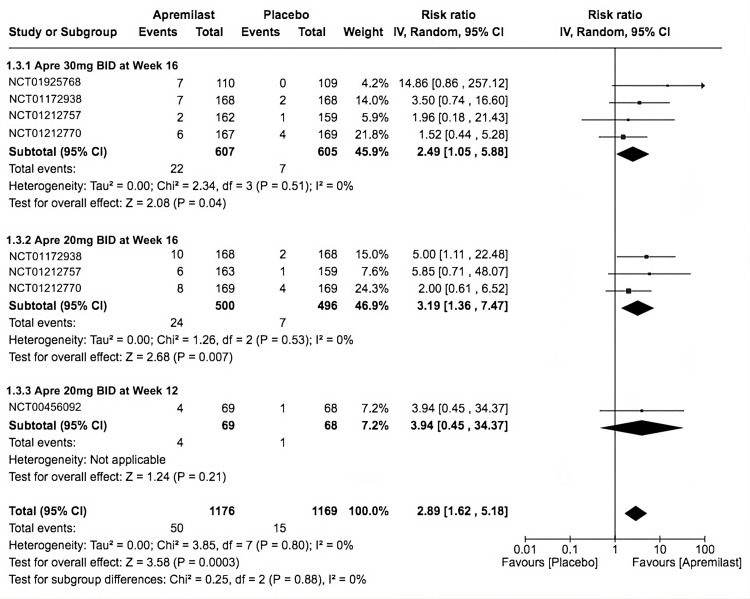
Forest plot of ACR70. Sources: [9-13]. CI, confidence interval; IV, inverse variance; RR, risk ratio; BID, twice a day; ACR70, American College of Rheumatology Response Criteria 70

The total apremilast ACR70 events in all trials had a higher occurrence than placebo (RR = 2.89, 95% CI 1.62-5.18; *P* = 0.0003; *I*^2^ = 0%). The second subgroup had a higher occurrence of ACR70 improvement that was statistically significant in favor of apremilast (RR = 3.19, 95% CI 1.36-7.47; *P* = 0.007; *I*^2^ = 0%), followed by the first subgroup (RR = 2.49, 95% CI 1.05-5.88; *P* = 0.04; *I*^2^ = 0%). Finally, the last subgroup had the highest RR, but it was insignificant (RR = 3.94, 95% CI 0.45-34.37; *P* = 0.21; *I*^2^ = not applicable; Figure 5).

HAQ-DI Mean Change From Baseline

**Figure 6:**
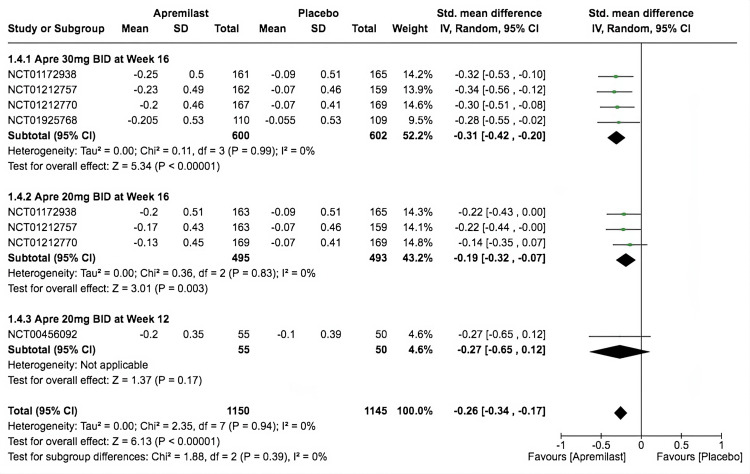
Forest plot of HAQ-DI. Sources: [9-13]. CI, confidence interval; IV, inverse variance; SMD, standardized mean difference; SD, standard deviation; BID, twice a day; HAQ-DI, Health Assessment Questionnaire and Disability Index

All five RCTs significantly favored apremilast compared to placebo in terms of the mean change from baseline in the HAQ-DI (SMD = -0.26, 95% CI -0.34 to -0.17; *P* < 0.00001; *I*^2^ = 0%). The first subgroup had the most significant score reduction from baseline (SMD = -0.31, 95% CI -0.42 to -0.2; *P* < 0.00001; *I*^2^ = 0%). Second was the following subgroup with an SMD of -0.19, 95% CI of -0.32 to -0.07, *P* < 0.003, and *I*^2^ = 0%. However, the third subgroup did not achieve a significant reduction from baseline (SMD = -0.27, 95% CI -0.65 to 0.12; *P* = 0.17; *I*^2^ = not applicable; Figure 6).

Any AEs

**Figure 7:**
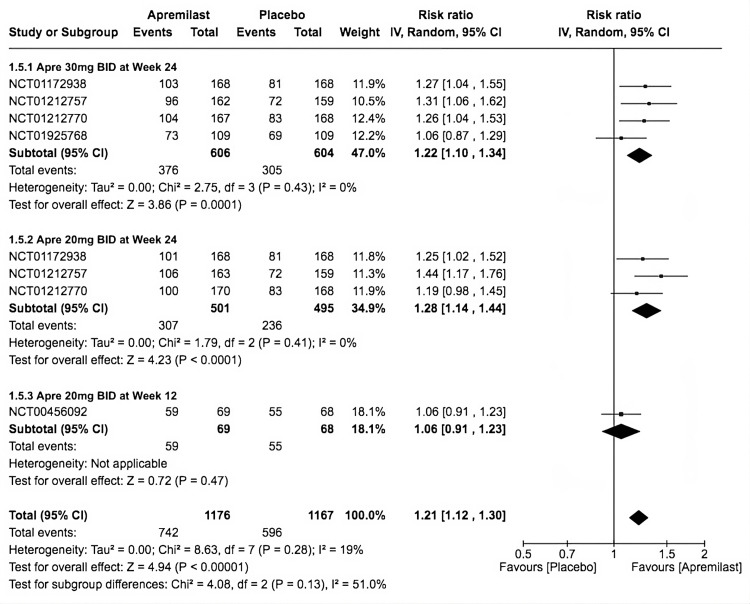
Forest plot of any adverse event. Sources: [9-13]. CI, confidence interval; IV, inverse variance; RR, risk ratio; BID, twice a day

The included trials showed a significant occurrence of any AEs with apremilast (RR = 1.21, 95% CI 1.12-1.30; *P* < 0.00001; *I*^2^ = 19%). To illustrate, most were non-serious gastrointestinal events like nausea, diarrhea, and vomiting. The second subgroup had the highest significant events (RR = 1.28, 95% CI 1.14-1.44; *P* < 0.0001; *I*^2^ = 0%), followed by the first subgroup (RR = 1.22, 95% CI 1.10-1.34; *P* = 0.0001; *I*^2^ = 0%). On the other hand, the third subgroup showed an insignificant difference between apremilast and placebo (RR = 1.06, 95% CI 0.91-1.23; *P* = 0.47; *I*^2^ = not applicable; Figure 7).

SAEs

**Figure 8:**
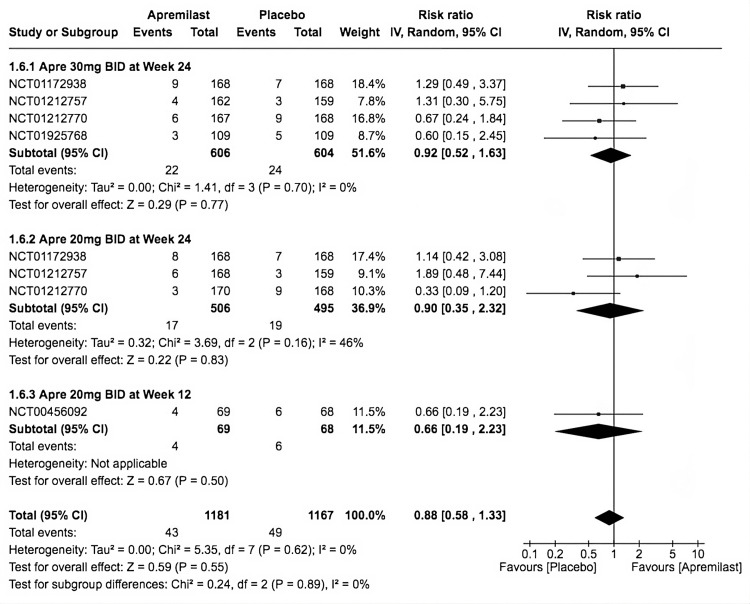
Forest plot of serious adverse events. Sources: [9-13]. CI, confidence interval; IV, inverse variance; RR, risk ratio; BID, twice a day

All five trials conveyed an insignificant difference between the incidence of SAEs in apremilast and placebo (RR = 0.88, 95% CI 0.58-1.33; *P* = 0.55; *I*^2^ = 0%). The first (RR = 0.92, 95% CI 0.52-1.63; *P* = 0.77; *I*^2^ = 0%), second (RR = 0.90, 95% CI 0.35-2.32; *P* = 0.83; *I*^2^ = 46%), and third subgroups (RR = 0.66, 95% CI 0.19-2.23; *P* = 0.50; *I*^2^ = not applicable) showed that apremilast did not cause a significant AE in any included trial (Figure 8).

Mortality

**Figure 9:**
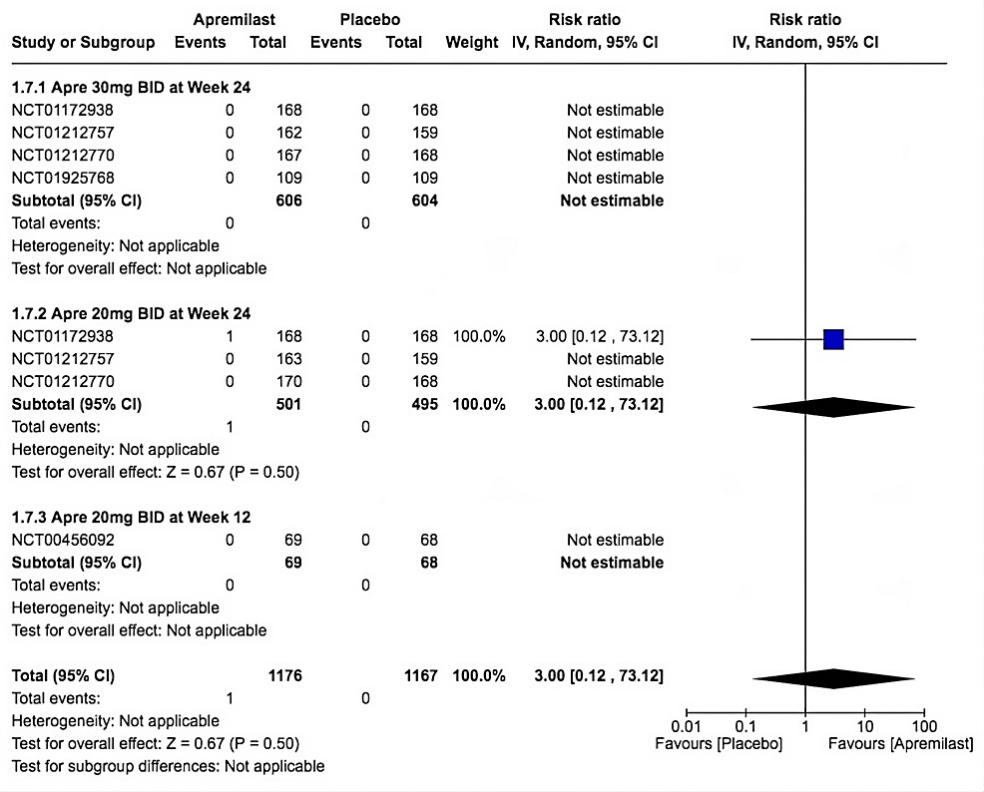
Forest plot of mortality. Sources: [9-13]. CI, confidence interval; IV, inverse variance; RR, risk ratio; BID, twice a day

Only one trial reported a mortality incidence in the apremilast arm within the second subgroup (RR = 3.00, 95% CI 0.12-73.12; *P* = 0.50; *I*^2^ = not applicable). Furthermore, the trial claimed that the cause was unlinked to apremilast since the patient developed multiorgan failure that was secondary to preexisting vitamin B12 deficiency. Neither the first nor the third subgroup reported an incidence of death; as a result, the analysis was not estimable (Figure 9).

Discussion

Methotrexate was the first DMARD to be frequently utilized in treating PsA due to its efficacy in treating skin and joint disorders [20]. Based on ACR20, ACR50, and HAQ-DI, active drugs, including methotrexate are more effective than placebo [21]. However, the use of methotrexate as monotherapy was shown to be less effective than combination therapy of both methotrexate and biologics [21]. Based on a study by Scarpa et al., it is demonstrated that the early use of methotrexate in PsA is effective in resolving clinical symptoms. However, much of the disease marker activity was not substantially affected [22]. In addition, as stated by Chandran et al., the use of higher dose and early initiation of the treatment yielded better outcomes in the radiological peripheral joint damage progression where the measured reduction in actively inflamed joints was around 40% [23].

In this systematic review and meta-analysis of five RCTs with a total of 1,849 participants, we assessed the efficacy and safety of oral apremilast compared to placebo in treating patients with PsA. The pooled estimate yielded a statistically significant improvement in ACR20, ACR50, ACR70, and HAQ-DI of PsA treated with either oral apremilast 20 mg BID or 30 mg BID compared to placebo.

The primary efficacy endpoint was based on the ACR-modified response criteria, which is defined as a minimum of 20%, 50%, and 70% improvement from baseline. Since its proposal in the early 1990s, it has been widely adopted in many clinical trials and is considered a standardized scale to assess the improvement in the tender joint count and swollen joint count [24]. In our study, patients who achieved ACR20 at week 16 showed that apremilast (RR = 1.92, 95% CI 1.66-2.21) is significantly more effective than placebo. The proportion of patients in our study who were on apremilast 30 mg (RR = 1.99, 95% CI 1.64-2.41) achieved higher response than those on apremilast 20 mg (RR = 1.71, 95% CI 1.37-2.13). According to a previous study, apremilast and numerous biologics have shown a better chance of scoring higher responses in ACR20 [24]. In a similar meta-analysis, the RRs for apremilast 20 and 30 mg versus placebo were 1.7 and 1.98, respectively [25]. Furthermore, according to Qu et al., apremilast 20 and 30 mg showed more significant scores statistically than placebo [26]. These results were consistent with our results, demonstrating greater efficacy of apremilast. In our study, significantly more patients achieved higher ACR50 and ACR70 responses than placebo (RR = 2.34 and 2.89, respectively). This was also demonstrated in a study by Kavanaugh et al. [10], wherein apremilast at 20 and 30 mg exhibited higher ACR50 and ACR70 responses compared to the placebo. Similarly, biologics, including apremilast, proved significantly more effective results compared to placebo in terms of ACR20 and 50% response rate [24,27].

The secondary efficacy endpoint of this study was the HAQ-DI change from baseline. Apremilast proved to be more effective than placebo in terms of HAQ-DI, with the most significant score reduction observed for apremilast 30 mg (SMD = -0.31, 95% CI -0.42 to -0.2; *P* < 0.00001). Similarly, according to Qu et al., apremilast 20 and 30 mg demonstrated significant improvement in terms of physical functions when compared to placebo, with SMD of -0.11 and -0.16, respectively [26].

Apremilast doses are well-tolerated and have a relatively good and acceptable safety profile on short-term follow-up [27]. The most commonly reported non-SAEs among patients were nausea, upper respiratory tract infections, and diarrhea, which occurred shortly after the initiation of the treatment course and were typically self-limited [24-26,28]. We assessed the safety of the drug by evaluating the rate of non-SAEs, SAEs, and mortality among the five included RCTs. In terms of AE rates, apremilast 20 mg BID had a higher rate than apremilast 30 mg BID. This was consistent with the findings of three previous studies in which the authors reported apremilast doses had higher rates of common non-SAEs than placebo [25-27]. In all included studies, only one death was reported in the apremilast 20 mg BID group; however, the results did not negatively impact the safety of the drug since it was unrelated to the administration of the drug.

Overall, apremilast 30 mg BID was found to be therapeutically more efficacious than apremilast 20 mg BID. Based on Qu et al., all the included trials in the study have shown a dose-dependent effect when treated with apremilast. However, in terms of efficacy, the difference between the doses was not statistically significant [26].

## Conclusions

In conclusion, the results demonstrated a statistically significant improvement in disease outcomes when utilizing apremilast across all efficacious endpoints, including HAQ-DI, ACR20, ACR50, and ACR70 scales. When compared to placebo, apremilast specifically bettered functionality although associated with an increased prevalence of non-SAEs, especially gastrointestinal symptoms such as nausea and vomiting. Serious side effects and mortality of apremilast were insignificant in comparison to placebo. In addition, the abated heterogeneity in the meta-analysis provided clarity in assessing the results. To our knowledge, no previous systematic review and meta-analysis was conducted to solely evaluate the efficacy and safety of apremilast, using ACR20/50/70 and HAQ-DI, when compared to placebo for the management of patients with PsA. The abated heterogeneity in the meta-analysis provided clarity in assessing the results. On the other hand, the included RCTs might have a bias risk that could influence the results. Finally, the number of RCTs included may have been restricted. Further trials, especially ones comparing apremilast to the current PsA management, are needed to improve its generalizability and validity.
